# Culture of Silence: Examining the Influence of Culture in Mock-Eyewitness Reports

**DOI:** 10.3390/bs16091540

**Published:** 2026-09-01

**Authors:** Alejandra De La Fuente Vilar, Lorraine Hope, Feni Kontogianni, Amunra Layal

**Affiliations:** 1 School of Criminology and Criminal Justice, University of Portsmouth, Portsmouth PO1 2BZ, UK; amunra.layal@gmail.com; 2 School of Psychology, Sport and Health Sciences, University of Portsmouth, Portsmouth PO1 2DY, UK; lorraine.hope@port.ac.uk; 3 School of Psychology and Social Sciences, University of Winchester, Winchester SO22 4NR, UK; feni.kontogianni@winchester.ac.uk

**Keywords:** culture, eyewitness, information gathering, reluctance, online crime reporting

## Abstract

Culture shapes communication norms and societal expectations about an individual’s ties to their social group. This research examined the extent to which cultural influences impact reporting of crime information, including when there is pre-existing relationship between the witness and perpetrator. In a pre-registered online quasi-experiment (*N* = 163), we examined mock-witness statements about a road traffic incident provided by individuals from the United Kingdom and China (fluent in English). For both groups of participants, the identity of the perpetrator was manipulated to simulate three levels of relationship proximity with mock-witnesses (close–same culture; stranger–same culture; stranger–different culture). In line with our hypothesis, we found an effect of cultural group on the amount of correct information reported. Replicating previous research, individuals from the UK, whose culture is associated with low-context communication preferences, reported significantly more information than those from China, who are associated with a high-context communication culture. Despite an increase in disclosure when reporting information about an out-group target, this affiliation effect was not statistically significant, and neither was the interaction effect. These findings highlight that witnesses’ cultural background should be considered in investigative interviewing contexts, and caution that a witness–perpetrator relationship, to a lesser extent, may jeopardise reporting of information by eyewitnesses. Limitations of the study and future research are discussed.

## 1. Introduction

In information-gathering contexts, the way culture shapes how eyewitnesses remember, interpret and report crimes can determine the extent to which they provide accurate and complete statements (Hope et al., 2022). However, cultural group affiliation may also affect reporting of crime information, considering the expectations regarding social bonds, group harmony and loyalty to those from the individual’s community, particularly in collectivistic cultures (Markus & Kitayama, 1991; Triandis, 1995). Across cultures, witness cooperation may be further affected when there is an existing relationship with the perpetrator as this can inhibit disclosure of incriminating information (De La Fuente Vilar et al., 2020), due to fear of retribution or avoidance of negative consequences (Papp et al., 2019; Spencer & Stern, 2001). Whilst there is small but growing evidence on the independent effect of culture on eyewitness statements (e.g., Anakwah et al., 2020a), the combined effect of an existing witness–perpetrator relationship on information disclosure across different cultures has not previously been directly examined, to our knowledge.

### 1.1. Witness Cooperation Decision Model

An individual’s rational decision to report a crime or not results from analysing the material and psychological benefits and costs of cooperating with the police (Kidd, 1979). Extending this model to witness cooperation in police interviews, it has been argued that a subjective evaluation of high costs with low benefits can lead to the decision to not fully cooperate with the police (De La Fuente Vilar, 2022), which decreases information disclosure (De La Fuente Vilar et al., 2020). Situational and individual factors can affect witness cooperation decisions, such as a relationship between a witness and the perpetrator (e.g., Papp et al., 2019). Specifically, disclosure of information in investigative interviews may be linked to particular moral judgements of the perpetrator.

*Relation regulation* in moral judgements is the evaluation of others’ (and one’s own) behaviour guided by prescriptive models of social relations (Rai & Fiske, 2011), with the motivation to maintain the relationship. So, an action (e.g., a crime) is not evaluated in isolation, but the social–relational context in which it occurs matters. Rai and Fiske (2011) proposed individuals judge the morality of an act based on four motivations: *unity*—prioritising support of the in-group; *hierarchy*—protecting those in superior ranks; *equality*—balanced treatment based on expected in-kind reciprocity; and *proportionality*—judging if the act is justified in terms of their overall costs and benefits. Consequently, if there is a witness–perpetrator relationship, the witness’ moral interpretation of the perpetrator’s actions will not occur in isolation (e.g., based on the crime alone), but rather the context of their social relationship may be considered.

Research in this arena so far has demonstrated that the decision to report incriminating information about a known perpetrator is more complex than reporting such information about a stranger. Victims and bystanders are more willing to report different types of crimes to the authorities and to intervene in violent situations when the perpetrator is unknown, compared to when it is a friend, due to strong peer group bonds (Nicksa, 2014; Schwartz & DeKeseredy, 1997; Tolsma et al., 2012). Related findings show that witnesses are more likely to provide an alibi for a suspect who is a friend or someone close to them (Kienzle & Levett, 2018), even if it is a false alibi (Marion & Burke, 2017). These effects of relationship on crime reporting have been argued to be founded in evolutionary principles of kin selection whereby help is offered to those with whom genetic material is shared (Hamilton, 1964). Extending beyond family relationships, reciprocal altruism explains these effects as ‘altruistic’ acts offered to individuals with whom we are socially related within a community or group, as they may reciprocate the altruistic behaviour in the future (Trivers, 1971).

The attitudinal bias in favour of someone from an individual’s ‘own group’ over members of other groups is also a well-established phenomenon, as a product of intergroup perception and competition (Brewer, 1979). In-group-out-group differentiation is formed on salient categories that can classify an individual as belonging to a group distinct from another, such as cultural identity. There is a tendency to preserve in-group solidarity and justify hostility and exploitation for members of out-groups (‘we-group’ vs. ‘others-group’), even under minimal competition conditions, and when gain for the in-group is at the cost of the out-group (Tajfel et al., 1971). In applied legal contexts, there are competing effects of *similarity–leniency* and *out-group punitiveness* in which a shared identity can lead to lenient judgement towards in-group members and harsher judgments towards out-group members. On the other hand, the ‘*black sheep effect*’ is described as harsher judgements against in-group members (vs. out-group members) if the incriminating evidence is strong, as it constitutes a heightened threat to the group’s positive image (Taylor & Hosch, 2004).

So far, there has been limited empirical research on the influence of group affiliation in investigative interviewing contexts. In relation to facilitating disclosure, suspects’ perceptions of group affiliation between interviewer and themselves as interviewees have been found to increase disclosure (Brimbal et al., 2019). However, there is a lacuna in the literature, as the effect of any affiliation between witnesses and perpetrators on reporting has not yet been investigated. The current research examines differences in reporting crime information when a perpetrator is someone close to the witness from a shared culture; a stranger from a shared culture; or a stranger from a different culture.

### 1.2. Culture and Communication in Investigative Contexts

Culture refers to shared meanings, practices, beliefs that operate within a particular environment (Wang, 2013). Such shared understanding can deeply influence how memory is represented, structured, processed, and expressed across individuals (Wang, 2021). Through ongoing socialisation, individuals internalise social and cultural norms, rules and values that condition the ways in which they interact and communicate (Triandis, 1995). Amongst the cultural factors affecting witness reports is the variation in distinct communication styles which shape interactions in investigative contexts (see Hope et al., 2022 for a review).

According to Hall (1976), communication based on explicit and direct messages in which meaning is transmitted in the content of the message is characterised as *low-context*. On the other hand, in *high-context* communication meaning is embedded in the social or physical context of the interaction making the message implicit and indirect. Gudykunst et al. (1996) contended that the cultural dimension *individualism–collectivism* (degree of cohesiveness between individuals and groups; Hofstede, 1980) also relates to such communication behaviour. In this vein, a tendency towards low-context communication has been observed as dominant in individualistic cultures, whereas high-context communication is predominant in collectivist cultures (Gudykunst et al., 1996).

In investigative contexts, low- and high-context communication styles have been theorised as potential drivers that account for differences in amount and type of information reported by crime witnesses (Hope et al., 2023). Specifically, Anakwah et al. (2020a) and de Bruïne et al. (2025) found that individuals from Sub-Saharan Africa, a high-context communication and collectivistic society, reported significantly fewer correct details in mock-crime reports than their counterparts from Northern Europe, a high-context communication and individualistic society. This pattern of apparent under-reporting crime details by mock-witnesses from a high-context communication culture was replicated by Hope et al. (2023), in a different cross-cultural comparison of Lebanon and the United Kingdom (UK hereafter). Beyond the laboratory and sharing even the country of origin in a multicultural setting, Weiss et al. (2025) have found that South Africans witnesses identified as White provide more detailed descriptions of crime offences than Black witnesses and Coloured witnesses^1^. Altogether, this body of research suggests that cultural factors affect the amount of information spontaneously reported by (mock-)witnesses. 

Culture may also indirectly affect the amount of information reported by influencing witnesses’ cooperation with respect to intentionally withholding or disclosing information. How culture impacts witness cooperation however has not received the same level of scrutiny, despite established links between cultural orientation (in line with individualism–collectivism) and the strength of group affiliation (Markus & Kitayama, 1991; Triandis, 1989, 1995).

### 1.3. Group Affiliation and Cultural Expectations

Another marked effect of being socialised in either individualistic or collectivistic societies is the construction of the self, as either more independent or interdependent to others in the group (Markus & Kitayama, 1991). These cultural self-constructs seem to emerge early in life (Wang, 2004) and go beyond self-perception with phenomenological correlates aligning individual behaviour to in-group norms (D. Cohen & Gunz, 2002). In individualistic societies where independence is valued, the societal expectation is to pursue personal goals, thus individuals act according to their self-interests, while in collectivistic societies individuals are seen predominantly as group members and are expected to prioritise group goals and emphasise group loyalty (Triandis, 1989, 1995). Moreover, individuals in collectivistic societies are expected to be loyal to extended social circles acting in the interest of the in-group and to maintain harmony with in-group members (Markus & Kitayama, 1991).

While the effect of group affiliation in information-gathering contexts has received limited scholarly scrutiny, the more specific influence of interpersonal relationships and their interaction with cultural expectations has been overlooked. Research on cultural differences in moral judgement suggests relevant implications for information gathering. Notably, Ding et al. (2025) used vignettes showing moral violations (e.g., including disloyalty towards an in-group member). In line with relation regulation (Rai & Fiske, 2011), Ding et al. (2025) found differences in moral judgement ratings, depending on the closeness of the social relationship (e.g., parents as transgressor, vs. salespersons). Critically, they found a culture effect—with further decreased harshness in judgements for ‘close’ transgressors by Chinese participants (vs. United States counterparts). The current research examined if this cultural group effect extends to mock-witness reporting in online contexts.

### 1.4. Online Crime Reporting

Online police reporting platforms have been implemented widely to improve efficiency, allowing reports for crimes 24/7 (Wells et al., 2023). In 2018, the UK opened a single online digital national police station, which includes crime reporting channels online. One of the benefits highlighted to citizen users is that reports are handled following the same procedures as if someone used other means of communication (e.g., in person, or by telephone), making it an effective and efficient reporting format whilst increasing convenience (www.police.uk). Research supports that if designed properly, these technologies can be found to increase public cooperation and engagement with the police thereby facilitating effective community policing (van der Giessen & Bayerl, 2022).

In line with the witness cooperation decision-making model proposed by De La Fuente Vilar (2022), online reporting may increase cooperation by reducing the perceived material costs of making a report (e.g., time, money). Another perceived benefit for reporting crimes using digital platforms is that such medium does not involve ‘contact’, ‘co-presence’, or an ‘interaction’ with a police officer (Wells et al., 2023). While this is not the case for all members of the public, avoiding physical encounter with the police may be preferable, for instance, by individuals who hold negative perceptions of the police (Tyler & Fagan, 2008). Conversely, online reporting may decrease cooperation if perceived as impersonal or distant, in turn reducing perceived procedural justice, especially in certain crime types (Jackson et al., 2025). The public acceptance, in particular from victims as users of these digital reporting tools, is influenced by communication style, design (i.e., user experience) and the police response—the human follow-up to the online crime reports (Jackson et al., 2025; Tatenko, n.d.; van der Giessen & Bayerl, 2022; Vitro et al., 2025).

### 1.5. The Current Research

The main aim of this research was to examine how cultural group and communication styles affect online crime reports by witnesses if there is an existing relationship with the perpetrator. A mock-witness paradigm was used to investigate the effects of the level of proximity of the witness–perpetrator relationship on the amount and accuracy of information by individuals from different cultural groups. Research participants were from the UK and China. Previous empirical evidence on the effects of cultural orientation have identified differences between Westerners and Easterners (D. Cohen & Gunz, 2002), with these two countries falling respectively as individualistic and collectivistic societies (e.g., Triandis, 1989; Oyserman et al., 2002), and individuals as more independent and interdependent in relation to their group affiliation (Wang, 2004). Furthermore, cultural metrics show that the UK and China differ in individualism scores, with UK scoring 76 while China’s score is 43 (The Culture Factor Group, 2023). The lower score for China indicates that culturally people act in the interests of the group, with cooperative and loyal relationships with those in the in-group, and cold or hostile attitudes towards out-group individuals. In contrast, in the UK, personal fulfilment is prioritised, with individuals having looser ties to the social group, in a ‘me’ culture focused on the individual (The Culture Factor Group, 2023). Furthermore, China is widely characterised as a high-context culture, where communication is more indirect and relational, whereas the United Kingdom is typically described as a low-context culture, favouring more explicit and direct communication styles (Hall, 1976; Ting-Toomey, 1999; Samovar et al., 2010).

Participants were asked to take on the role of a witness to a traffic road incident and provide an online mock-report to the authorities. The perceived proximity of the relationship between the witness and the perpetrator was manipulated altering the identity of the driver causing the accident by either knowing them personally or not and sharing their culture or not. Participants were asked to provide an online mock-witness report of the incident in which the perpetrator was either someone *close* to them from their *same culture* (close–same culture), a *stranger* from their *same culture* (stranger–same culture)*,* or a *stranger* from a *different* culture (stranger–different culture). The report consisted of a free report followed by follow-up questions with multiple choice answers.

The research hypotheses were pre-registered on As Predicted (https://aspredicted.org/thsk-9pdg.pdf accessed on 17 August 2026). Specifically, we hypothesised a main effect of proximity of the relationship to the perpetrator on information disclosure (Nicksa, 2014; Tolsma et al., 2012), such that less information would be reported in the close–same culture condition relative to both the stranger–same and stranger–different culture conditions (Hypothesis 1); and in the stranger–same culture relative to the stranger–different culture condition (Hypothesis 2). Based on past research by Anakwah et al. (2020a) and Hope et al. (2023), we predicted an effect of cultural group on information disclosure, such that less information is reported by participants from the high-context communication condition (China participants) in comparison to the low-context communication condition (UK participants) in free recall accounts irrespective of proximity conditions (Hypothesis 3).

Additionally, as a secondary aim we explored whether cultural group influences the use of communication strategies to provide and withhold information (to protect the perpetrator) as expressions of cooperative–uncooperative behaviour. Past research on counterinterrogation tactics by Alison et al. (2014) defined as deliberate strategies to resist cooperation with the police has shown that uncooperative interviewees often use verbal strategies that represent an attempt to appear cooperative by providing information (that is known); passive behaviour such as silence and gaze avoidance; passive verbal behaviour, which represents limited answers and claims of lack of memory; no comment utterances; and retractions in which there is a withdrawal of a statement previously made.

Other measures of interest were individual and cultural variations in direct communication style (Park et al., 2012), and cultural orientation (measured by the Converging Measurement of Horizontal and Vertical Individualism and Collectivism; Triandis & Gelfand, 1998). We also explored potential influences of police perceptions on witness reports given previous research demonstrating a relationship between perceptions of police legitimacy and trust with cooperation with the police (Tyler & Fagan, 2008), which critically seem to be influenced by cultural and political contexts (Cao et al., 1998; McCarthy et al., 2022; Tankebe et al., 2016).

## 2. Method

### 2.1. Design and Sample

The study followed a quasi-experimental method, with a 2 (cultural group: United Kingdom [UK] vs. China) × 3 (proximity of relationship: close–same culture; stranger–same culture; stranger–different culture) partially crossed factorial design. Naturally occurring groups based on self-identified ethnicity were used for cultural group, whereas for proximity of relationship, participants were assigned to just one condition. Allocation was based on participants’ responses to a request to name a ‘target’ person for a seemingly unrelated task. Participants named one person who was ‘important’ to them, with whom they share a ‘strong and close relationship’ and have known for ‘several years’. When participants chose a target person from a shared culture, participants were randomly assigned to any of the three conditions. Assignment occurred automatically into the stranger–different culture condition if the target person chosen was from a different culture from the participant. A fully crossed design was not selected for reasons of sample accessibility, selecting instead to examine the comparisons of most applied relevance. The research project was approved by two institutional research ethics boards and all human participants provided informed consent.

An a priori G*Power 3.1.9.7 statistical analysis (Faul et al., 2007) showed that a sample of 206 participants was required for a 90% chance of detecting a conventional medium effect size *f* = 0.25, based on previous related findings on cultural effects on witness reports (Anakwah et al., 2020a, 2020b; Hope et al., 2023). Pre-registrations of the sample size calculation and primary analyses are available on As Predicted (https://aspredicted.org/thsk-9pdg.pdf accessed on 17 August 2026). Whilst data collection was terminated with 208 responses, there were 45 exclusions for different reasons. The inclusion criteria were: (i) being 18 years old and above, (ii) being British or Chinese (self-identified ethnicity and/or country of origin was used as proxy for cultural background), (iii) being able to fluently read and understand English, and (iv) not failing more than one of three attention checks.

All participants fulfilled the age requirement. Participants provided their ethnicity and country of origin in open responses to allow for self-identification, which were used to confirm cultural group. Participants’ country of origin was used as proxy for cultural group in the UK sample, leading to 14 exclusions. All included participants’ country of origin is the UK (responses included ‘UK’ as well as ‘England’, ‘Great Britain/Britain’, and ‘Wales’). Ethnicity responses alone could not be used to confirm cultural group as many responses included only a racial descriptor (‘White’/’Caucasian’). However, when ethnicity responses were examined, 10 participants were excluded as they were from the UK but identified as other than British culturally (responses included ‘Asian’, ‘British-Asian’, ‘British Indian’, ‘Vietnamese’, ‘Bangladeshi’, and ‘Mixed’). For the Chinese sample, two participants were excluded on this criterion. Participants’ responses to ethnicity and/or country of origin were used as a proxy for cultural group in the Chinese sample. If ethnicity was self-described as ‘Chinese’, ‘Han Chinese’ participants were included even when their country of origin was not China. If ethnicity was self-described as ‘Asian’, ‘East Asian’, or others, participants were included only if their country of origin was ‘China’.

Research has shown that reporting crime information in a second language can reduce the amount and accuracy of information disclosed (Ewens et al., 2016). To confirm English language proficiency, we used sample tests (e.g., *Test Your English*©) publicly available online: https://www.cambridgeenglish.org/test-your-english/general-english/ (accessed on 17 August 2026; Cambridge University Press & Assessment, 2026). The test is not a direct measure of English language proficiency; however, a score of 18/25 corresponds to the B2 level of the Common European Framework of Reference for Languages (Council of Europe, 2020) and was applied as the eligibility criterion for English proficiency, which led to exclusion of eight participants. Lastly, four participants were excluded for failing data quality using attention checks, and seven participants were excluded due to attrition.

The final sample were *N* = 163 (*n* = 85 UK and *n* = 78 China) participants, with the following distribution across the three conditions: *n* = 41 for close–same culture; *n* = 31 for stranger–same culture; *n* = 91 for stranger–different culture. The final unbalanced group sizes were a trade-off to increase the plausibility of the research paradigm based on the participants’ responses regarding the target person (De La Fuente Vilar et al., 2023); however, statistical corrections were needed (see Section 3.1. *Preliminary Analyses*). There is a significant difference (*p* < .001, *d* = −0.81) in participants’ age, where UK *M*_age_ = 23.38 and *SD* = 11.56 for age range 18–70 years, while China *M*_age_ = 32.00 and *SD* = 9.69 for age range 19–61 years. There is also a significant but small difference in gender distribution across cultural groups (*p* = .022, Cramer’s *V* = 0.22), with 80.72% female, 16.87% male and 2.41% non-binary in the UK sample, and 61.54% female, 35.90% male and 2.56% non-binary in the China sample.

The pool of participants was mixed. Participants were local students and from the community in a small-to-medium-sized city in the UK (*n* = 91) and were recruited through Prolific (*n* = 72). Data collection started locally but after encountering difficulties recruiting the required sample size of Chinese participants it was moved to Prolific (https://www.prolific.com).

### 2.2. Materials

#### 2.2.1. Multimedia Stimuli Package

To set the context of the incident, a set of scenario-specific images was used, and participants were asked to imagine themselves being in their living room at home when they heard increasingly loud traffic noise through the window. They were then instructed to imagine they stepped out to their terrace, located on the second floor from which there is an elevated view of a road traffic incident; in this way participants had the same first-perspective point of view as the video stimulus event.

The video showing the incident lasts 2′30″ and is publicly available on YouTube (Vance, 2013). The scenario depicts a traffic incident in which a black car attempts to flee after an initial collision, with the driver not visible. A taxi driver, from the first car hit, exits his vehicle, confronts the black car, and strikes its window while standing in its path. The black car then manoeuvres erratically, colliding with multiple vehicles before accelerating away. The road traffic incident depicted in the stimulus video does not portray people suffering from any injuries, only minor material damage to vehicles. This type of incident was deliberately selected to manage believability in the research paradigm as some participants needed to imagine the perpetrator was someone close to them. Secondly, witnessing such traffic incidents is a plausible experience for participants living in cities globally (World Health Organization, 2023). Furthermore, the methodological choice of a victimless crime alleviates some of the potential cultural effects affecting the perceptions of the severity of other types of crimes (e.g., Buchtel et al., 2015).

The stimuli package included images to situate participants in a shared legal and investigative context in the experiment. Participants were informed that failure to stop and report the collision that caused damage to other vehicles is a punishable offence by law. Whilst this is not universal, it is the case under the Road Traffic Act 1988, s. 170 in the UK. Participants were informed of three further traffic violations following the witnessed incident which were committed by the driver of the black car, including repeatedly exceeding the speed limit, using their mobile phone whilst driving, and driving past a red traffic light, which they were informed legally constituted careless and dangerous driving (UK Government, 1988, ss. 2–3). Additionally, an image mock-up of television coverage was shown with the news headline speculating the driver was under the influence of alcohol. Lastly, participants were informed of the consequences for the driver if found, such as a large fine, penalty points on their driving licence and the potential of being disqualified from driving. All materials are publicly available on the Open Science Framework project’s page https://osf.io/592re/overview?view_only=8c48b6fe5f8048b2b922a8aa7dcc4b43 accessed on 17 August 2026).

#### 2.2.2. Questionnaires

To examine cultural orientation on the individualism–collectivism dimension across groups, we used the scale developed by Triandis and Gelfand (1998) as it can show differences in individualism and collectivism and horizontal and vertical aspects of each. The scale includes 16 items which assess respondents’ views regarding social relationships with responses ranging from low (1) to high agreement (9). Unlike previous conceptualisations of individualism and collectivism as a dichotomy, Triandis and Gelfand (1998) propose to also consider vertical and horizontal relationships that are about acceptance of hierarchies, or equality leading to four subscales: horizontal individualism (high on self-reliance), vertical individualism (high on competition and hedonism), horizontal collectivism (high on sociability and interdependence but not on family integrity) and vertical collectivism (high on family integrity and sociability and low on emotional distance from in-groups).

In addition, Park et al.’s (2012) measure of 6 items about direct communication style was used, in which agreement was rated on a 5-point Likert scale (1 = strongly disagree, 5 = strongly agree). Face needs can also affect the motivation to predominantly use a direct communication style; thus, Park et al.’s (2012) scale was used to measure face needs using the same Likert scale. Indirect communication is generally regarded as more face-saving. Empirical evidence supports that individuals from collectivistic contexts are more responsive to threats to positive face, referring to the desire to be approved by others and need to protect self-image, whereas those from individualistic cultures are more sensitive to threats to negative face that focuses more on protecting autonomy and freedom from imposition (see Park et al., 2012 for a review).

Participants also completed a post-report questionnaire which measures attitudes towards and perceived legitimacy of the police (Tyler & Fagan, 2008). The scale includes 27 items, with responses ranging from low (1) to high agreement (5), assessing legitimacy by measuring the aspects of obligation to obey the police; attitudes of trust and confidence; and identification with the police (i.e., sharing their values) (see this exploratory analysis in Supplementary Materials). Participants were also asked to indicate which verbal strategies they used in their online witness report in order to protect the perpetrator.

### 2.3. Procedure

Participants had the opportunity to read the participant information sheet and provided consent digitally. They provided basic demographic information in an open text format, which included: age, gender, ethnicity, country of origin, country of residence, native language. If they were not native English speakers, they took the English proficiency test. Next, participants were asked to provide the name of a target person. Specifically, they were asked to provide the name of someone important to them that they have known for several years with whom they share a strong and close relationship. It was noted that the person could be a friend or a family member. After they provided their name, this name (e.g., ‘X’) was automatically embedded in the rest of the questions they were asked to establish the cultural background of the target person. Participants had to self-identify whether the target shared the same culture as them (e.g., Is ‘X’ from the same culture as yours? Yes/No). Participants also provided information about the nature and length of their relationship with the target person.

After, participants were informed about their role as a witness to a road traffic incident. Participants received a multimedia stimuli package containing images to set the context of the incident before it took place. They were then instructed to pay attention and were told that they would be asked questions about the incident later. Participants watched the stimulus video and images in a slide show presentation. Then, participants were informed the incident was being investigated by the police and they were serving as a witness.

Participants were notified the person driving the black car (i.e., the perpetrator) was responsible for the road traffic incident. According to their allocated condition for proximity of relationship, the identity of the perpetrator was manipulated. Participants in the close–same culture condition were told they knew who was driving the car and identified the target person ‘X’ as the perpetrator, using the name they had previously provided. They were also reminded of their relationship to them (‘The driver was X who shares the same culture as you with whom you have a strong and close relationship’). Participants in the stranger–same culture condition were told they did not personally know the driver, but they did know the driver was from the same culture as the participant. Participants in the stranger–different culture condition were told they did not personally know the driver, but they did know the driver was from a different culture from the participant.

There was a manipulation check to confirm they understood the identity of the driver, which all participants passed. Participants were asked: Who was the driver of the black car? Lastly, before providing an incident report the instructions read: ‘The information you report about the incident will be used to further investigate this incident and ultimately to hold the driver of the black car responsible’. For the close–same culture condition, the name of the previously identified target person was used instead of driver. Additionally, all participants were explicitly instructed that they could not completely deny that they were witness to the event; however, they could choose what information to provide (‘In your report, you cannot completely deny that you witnessed the incident because the police know you saw the whole incident. It is up to you to decide which information to provide to the police’). Then participants were provided with unlimited space for a free report labelled ‘Incident Report’.

The following section had the same preface regarding information management as above and then included five follow-up questions regarding the incident that related to the driver’s level of responsibility and extent of the damage of the incident, such as ‘How did the incident start?’, ‘Which cars were involved in the incident?’. Each question offered the witness four potential responses of which they could select one as their answer. The responses offered were designed such that each response offered an increased number of details in response to the question. For example, the question ‘How did the incident start?’, had the following response options: (a) ‘I don’t know’, (b) ‘A car bumped into another car’, (c) ‘A black car bumped into the car in front of them’, or (d) ‘A black car bumped into the white taxi in front of them’. Participants then received questions about the verbal strategies they used when completing the incident report. Finally, participants completed the different questionnaires regarding their reports, perceptions of law enforcement, cultural orientation and communication style. They were thanked for their participation and received written debriefing explaining the purpose of the study in further detail.

### 2.4. Coding

Participants’ incident reports in the free report section were coded by two trained coders. Coders were an undergraduate psychology student and a postgraduate forensic psychology student who were trained by the first author, experienced in this type of data and coding. Coders were blind to the study’s conditions and hypotheses. Training included the provision and 2 h explanation of the coding scheme, in which all the details were coded in five-second segments of the stimulus video. Details were only coded when they were correct against the stimulus video. This coding represents a deviation from the pre-registration as hypotheses were formulated in terms of information rather than correct information, which is the main dependent variable of interest in this mock-witness paradigm. There was no hypothesis however regarding accuracy that would require coding of both correct and incorrect details (to predict reporting of erroneous information from memory).

For instance, the following excerpt of a report would tally five correct details: ‘A white (1) van (1) and a grey (1) car (1) drive by (1)’. Details mentioned were only coded once if reported in the same segment [e.g., ‘The taxi (1) driver (1) stood (1) in the middle of the road (1)’. The taxi driver (0) had a bat (1)]. Subjective comments were not coded (e.g., ‘He looked very angry’). The coding training also included an exemplar of a coded transcript, and a joint practice coding exercise in which coders received individual feedback. Training was followed by regular calibration meetings in which doubts from batch transcript coding were discussed and any discrepancies were resolved through discussion as a group, leading to rule setting and re-coding iteratively. The last author, who received the same training and was also blind to the conditions and hypotheses, coded a randomly selected subset of transcripts (*n* = 40, ~25%). To establish inter-rater reliability, a two-way mixed-effects intraclass correlation coefficient (ICC) for absolute agreement was calculated. The ICC indicated excellent levels of inter-rater agreement, ICC = 0.98 (95% CI [0.97, 0.99]), suggesting a high degree of consistency across coders (Koo & Li, 2016).

Participants’ answers to the follow-up questions were tallied based on the number of details included in the predetermined multiple-choice responses to transform that data into a continuous variable for analysis. Each answer included one to four details, making 20 the maximum total number of details available to choose to report in the follow-up questions. The analysis of the qualitative data from post-incident questionnaires deviated from the plan of conducting deductive analysis following the categories proposed for interview data by Alison et al. (2014) as the data did not correspond with those verbal strategies. Instead, a data-driven content analysis was conducted with codes derived inductively from the data, refined in an iterative process between the first and last author. The last author was the main coder, and all discrepancies were resolved through discussion.

## 3. Results

### 3.1. Preliminary Analyses

Participants reported high levels of agreement regarding finding the research scenario realistic (*M* = 5.74, *SD* = 1.18), and self-reported high engagement with the witness role (*M* = 6.09, *SD* = 1.06). All participants provided either a name or similar identifier for their target person (e.g., ‘mum’). Descriptive analysis shows that participants mostly chose a member of their family as the target (46%), with the rest of the participants evenly split between choosing their romantic partner (27.6%) or a friend (26.4%). Participants’ relationship length was on average *M* = 15.21 years (*SD* = 10.52), from which it can be inferred close proximity to the selected target persons across participants.

Inspection of data normality prior to analyses showed that the main dependent variable, total number of correct details reported, was positively skewed. Following Field (2018), a correction was applied using a square root transformation, resulting in acceptable normality (skewness = 0.24). Levene’s test for equality of variances on the transformed dependent variable was not statistically significant (*p* = .357), indicating the assumption of equal variances is not violated; thus, all subsequent analyses were conducted using that transformed variable for number of correct details during free recall in the mock-witness reports.

### 3.2. Confirmatory Analyses

We pre-registered the examination of main effects of cultural group and proximity of the (witness–perpetrator) relationship on information disclosure in the incident free report (open response format), followed by the analysis of an interaction effect, and if significant, we planned to follow up the effects of the two levels of cultural groups on each type of proximity group [low communication (UK) vs. high communication (China) on close–same culture, close–different culture, stranger–different culture].

**Figure 1:**
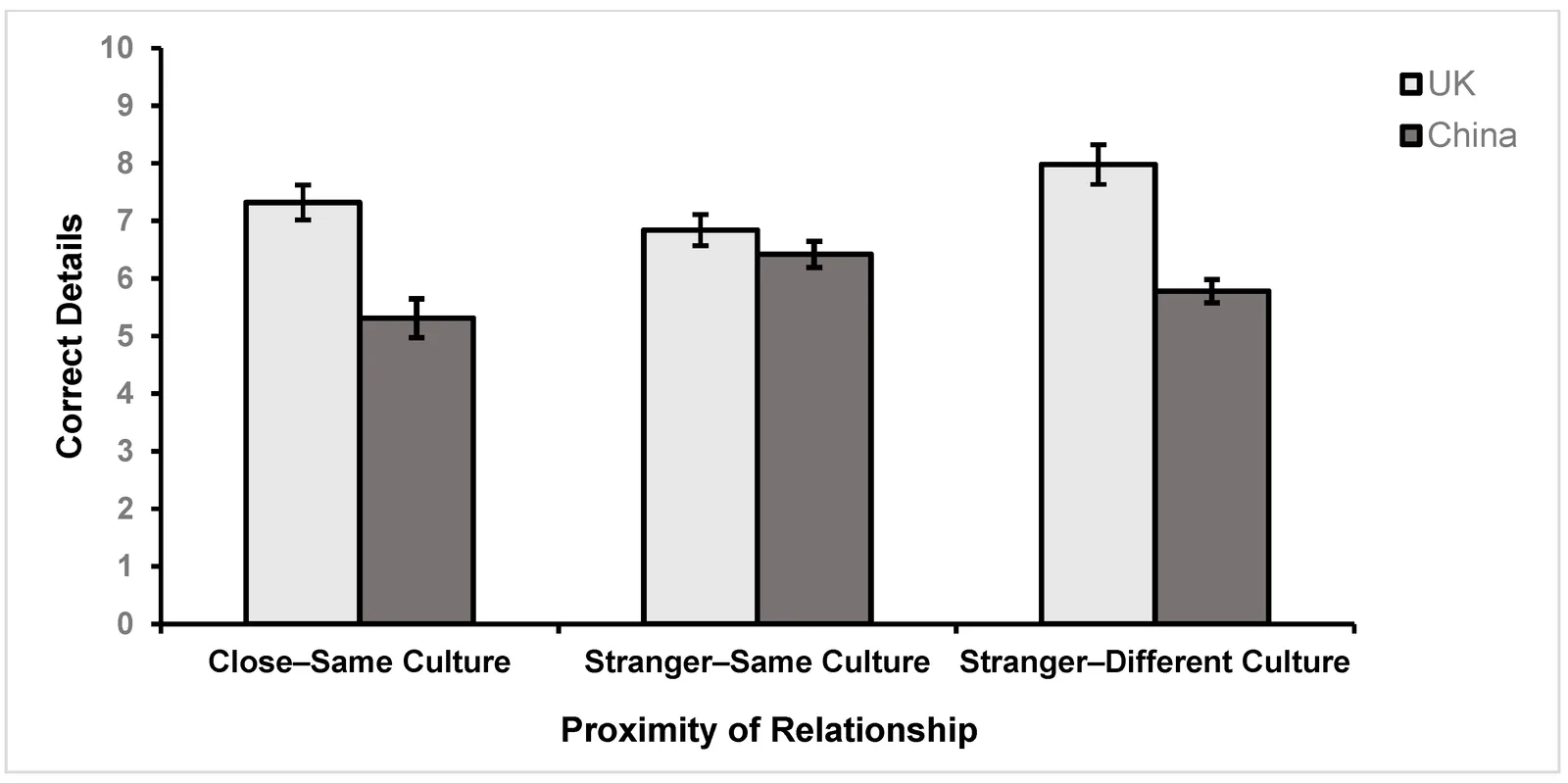
Correct details by condition in the free report. *Note*: Estimated marginal means of correct details reported spontaneously by participants from the UK and China are shown for the proximity of relationship conditions. Error bars represent ±1 standard error (SE).

A two-way analysis of variance (ANOVA) was conducted using the estimated marginal means of the data, which were square root transformed to correct for non-normality. The main and interaction effects of proximity of relationship and cultural group on free reporting were examined. The main effect of proximity of relationship was not significant, *F*(2, 157) = 1.67, *p* = .192, ω^2^ = .01, 95% CI [0.00, 0.05]. Estimated marginal means (EMMs) of the dependent variable for each group are back transformed from the square root transformation for interpretability and included in Figure 1.

Although we did not find support for Hypotheses 1 and 2, descriptive statistics partially showed the expected pattern of reporting. Participants reported the least number of correct details in the close–same culture condition (*EMM* = 6.32, *SE* = 0.25) followed by reporting in the stranger–same culture condition (*EMM* = 6.63, *SE* = 0.48), and with the largest amount in the stranger–different culture condition (*EMM* = 6.88, *SE* = 0.17). There is a large variance of reporting within conditions leading to an overlap across the two stranger conditions, which likely explains the lack of statistical significance despite the pattern observed (see Figure 1).

The ANOVA revealed a main effect of cultural group, *F* (1, 157) = 16.42, *p* < .001, ω^2^ = .09, 95% CI [0.02, 0.18], providing support for Hypothesis 3. Participants from the UK (*EMM* = 7.38, *SE* = 0.17) reported significantly more correct details than participants from China (*EMM* = 5.84, *SE* = 0.34) in the free recall mock-incident report.

Whilst an interaction between cultural group and proximity of the relationship was not predicted, the two-way ANOVA was conducted for completeness. The combined effect of cultural group × proximity of the relationship on correct information in the free report was not significant, *F*(2, 157) = 1.54, *p* = .219, ω^2^ = .07, 95% CI [0.00, 0.04].

### 3.3. Exploratory Analyses

#### 3.3.1. Effects of Cultural Group and Proximity of Relationship on Follow-Up Questions

**Figure 2:**
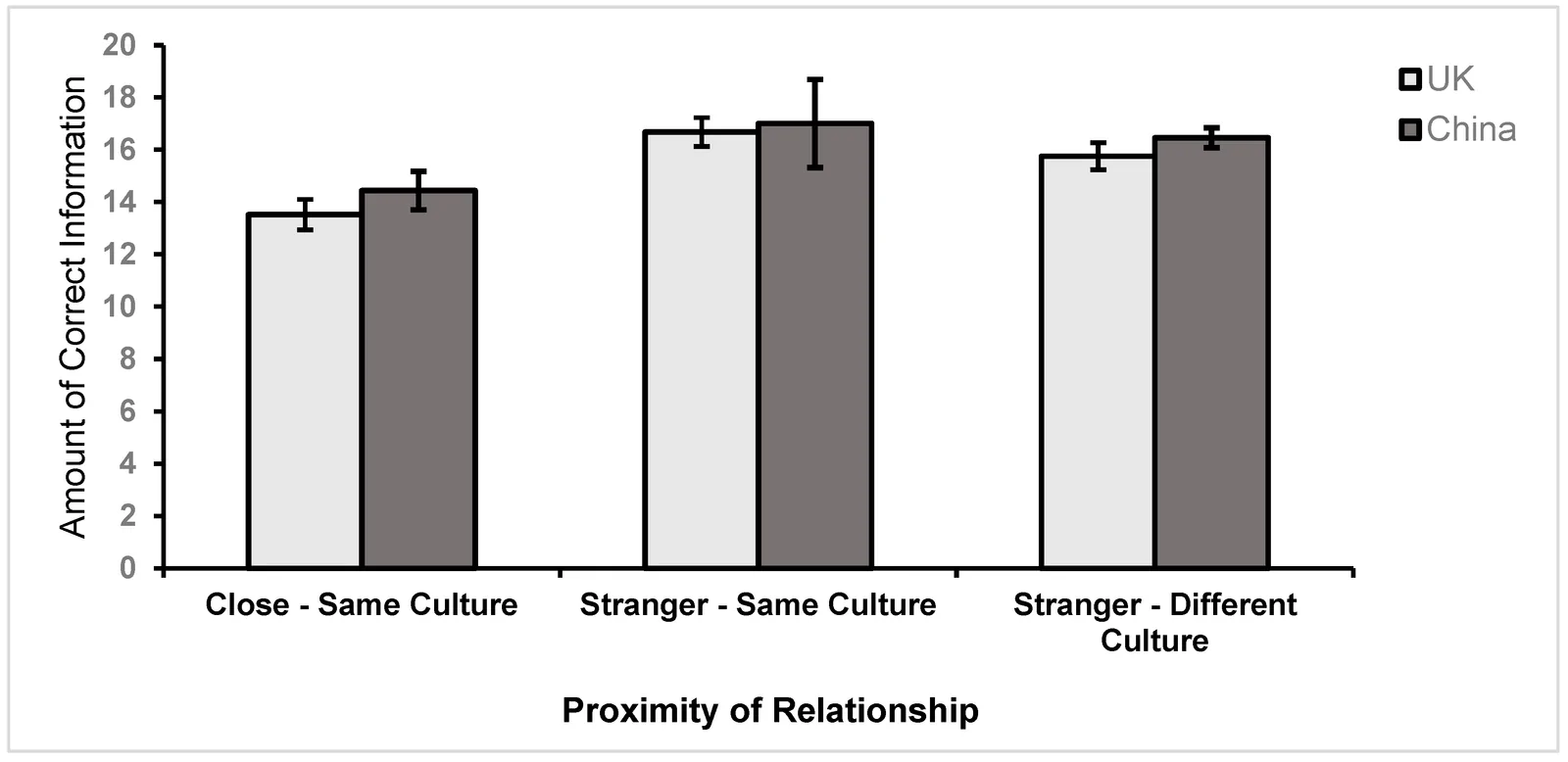
Amount of correct information by condition in the follow-up questions. *Note*: Means of information reported in follow-up questions by participants from the UK and China are shown for the proximity of relationship conditions. Error bars represent ±1 standard error (SE).

A continuous variable was computed to represent the information selected by participants in response to the follow-up questions. A one-way independent ANOVA revealed a significant effect of proximity of relationship on the amount of information reported in follow-up questions, *F* (2, 160) = 11.25, *p* < .001, ω^2^ = .11, 95% CI [0.03, 0.20]. Post hoc comparisons with Bonferroni corrections showed that participants in the close–same culture (*M* = 13.88, *SD* = 2.87) chose to report significantly less information than those in the stranger–same culture (*M* = 16.71, *SD* = 2.76, *t* (160) = −4.08, *p* < .001, *d* = −0.97, 95% CI [−1.56, −0.38]), and those in the stranger–different culture (*M* = 16.21, *SD* = 2.98, *t* (160) = −4.25, *p* < .001, d = −0.80, 95% CI [−1.27, −0.33]) conditions (See Figure 2).

The effect of cultural group on the information reported in the follow-up questions was examined. A Welch’s independent *t*-test was conducted as the data were not normally distributed (Shapiro–Wilk, *p* < .001). There was a non-significant main effect of cultural group, *t*(160.69) = −1.38, *p* = .169, *d* = −0.22, 95% CI [−0.52, −0.09], on the information participants chose to report in the follow-up questions. Participants from China (*M* = 16.06, *SD* = 2.87) reported more information, but not statistically different than those from the UK (*M* = 15.40, *SD* = 3.27).

#### 3.3.2. Communication Strategies by Condition

Participants provided agreement ratings regarding their use of four different verbal communication strategies in their free report (see Table 1 for descriptive statistics and Supplementary Materials for full reporting of inferential tests examining the relationship between use of communication strategies and free reporting). Overall proximity of the relationship seems to influence the use of providing a detailed report (i.e., increased proximity, decreased use), and a close relationship (vs. stranger) increases the use of providing only partial information. There is also an increased use of ‘partial information’ amongst Chinese (vs. UK) participants.

Additionally, participants were also asked to report in their own words which strategies, if any, they used for selecting which information to report and withhold in their free reports (see Table 2 for an overview of the self-reported communication strategies and Supplementary Materials for full reporting of the qualitative data analysis).

The most frequently used strategy across both cultural groups was related to providing a valid report based on their memory of the event. This strategy was also frequently used across proximity of relationship conditions, but more frequently used by those providing the incident free report about an unknown perpetrator without a shared cultural identity. Those in such conditions, like stranger–different culture, also reported higher use of simply providing all the information available.

Some participants also reported strategically omitting information in their free reporting of the incident, especially in the British sample. The proximity of the relationship, however, did not provide a clear pattern of use of this strategy. Instead, results indicate that the importance of the fictitious witness–perpetrator relationship increased the use of providing inaccurate information for those in the close–same culture condition, in order to portray the innocence of the driver of the black vehicle (i.e., participants’ target person) or by placing blame on another driver. Lastly, reporting important and incriminating information to help the police or given the severity of the incident was most frequently considered amongst British than Chinese participants.

#### 3.3.3. Direct Communication Style and Face-Saving Needs by Cultural Group

Differences in direct communication style scores were examined by cultural group. A composite score of the direct communication scale (Park et al., 2012) showed good reliability (Cronbach’s α = .81; Field, 2018) and was used as the covariate. A Welch’s independent *t*-test was conducted as the data were not normally distributed (Shapiro–Wilk, *p* < .001). There are no statistically significant differences in direct communication style scores between the cultural groups, Welch *t* (159) = −0.42, *p* = .678, *d* = −0.07 (95% CI −0.37, −0.24). Participants from the UK (*M* = 4.17, *SD* = 0.52) and from China (*M* = 4.20, *SD* = 0.53) had similar mean scores in the scale measuring direct communication style.

An analysis of covariance (ANCOVA) was carried out to examine the influence of cultural group and communication style on free reporting. There was a significant effect of cultural group on free reporting after controlling for the effect of direct communication style, *F*(1, 160) = 46.21, *p* < .001, ω^2^ = .21, 95% CI [0.11, 0.32].

In addition, face-saving needs were measured using Park et al.’s (2012) global score, which showed good reliability (Cronbach’s α = .83; Field, 2018). An independent *t*-test confirmed statistically significant differences in face-saving needs between cultural group conditions, *t* (161) = −2.03, *p* = .044, *d* = −0.32 (95% CI −0.63, 0.01). Average scores by participants from China (*M* = 16.66, *SD* = 1.80) were significantly higher than their UK counterparts (*M* = 16.12, *SD* = 1.63).

An ANCOVA was carried out to examine the influence of cultural group and face-saving needs on free reporting. There was a significant effect of cultural group after controlling for the general effect of face-saving, *F*(1, 160) = 48.49, *p* < .001, ω^2^ = .22, 95% CI [0.12, 0.33].

#### 3.3.4. Individualism and Collectivism Differences

Individual independent *t*-tests were run to examine comparisons by cultural group in subscale scores in the individualism and collectivism measure used (Triandis & Gelfand, 1998). There were significant differences on vertical individualism, *t* (161) = −2.68, *p* = .008, *d* = −0.42, 95% CI [−0.73, −0.11], with significantly higher average scores for participants from China (*M* = 5.32, *SD* = 1.53) than their UK counterparts (*M* = 4.68, *SD* = 1.52). Additionally, there were significant differences on horizontal collectivism, *t* (161) = 3.52, *p* < .001, *d* = 0.55, 95% CI [0.24, 0.86]. Average scores from participants from the UK (*M* = 6.96, *SD* = 1.08) were significantly higher than their counterparts from China (*M* = 6.33, *SD* = 1.22). However, there were no significant differences by cultural group on scores in horizontal individualism, Welch’s *t* (161) = −1.78, *p* = .077, *d* = −0.28, 95% CI [−0.59, 0.03], and vertical collectivism, Welch’s subscales, *t* (156.69) = −1.21, *p* = .230, *d* = −0.19, 95% CI [−0.50, 0.12] (see Supplementary Materials for further exploratory analyses).

## 4. Discussion

This research empirically examined for the first time how cultural group and an existing relationship with the perpetrator affect reporting by mock-eyewitnesses in an online format. First, we found some cultural differences affecting spontaneous reporting and disclosure of incriminating information in follow-up questions. Individuals from China, with a high-context communication and collectivistic culture, spontaneously reported significantly less information than their counterparts from the UK, a low-context communication and individualistic culture, regardless of the proximity of the relationship between the mock-witness and perpetrator. This finding is in line with our pre-registered hypothesis (H3) and replicates prior work showing that witnesses from high-context cultures disclose less information in free reporting formats relative to witnesses from low-context cultures (Anakwah et al., 2020a, 2020b; de Bruïne et al., 2025; Hope et al., 2023; Weiss et al., 2025).

Culturally those socialised in low-context societies, such as the UK, learn to communicate meaning directly in a message (Hall, 1976), which could explain why this group reported significantly more details. Qualitative data analysis supports this potential explanation. In comparison to Chinese participants, British participants reported more frequently that they relied on their memory to recall the incident and were motivated to provide important and incriminating information due to the incident severity. Conversely, as meaning is implicit in high-context communication, some information is inevitably lost. Chinese participants, in relation to British participants, acknowledged more frequently reporting only partial information.

It is also important to note that the influence of culture was only significant in free spontaneous reporting of the incident and did not hold up in the follow-up questions. Participants from China reported more information than participants from the UK, in a forced-responding question format. Recently, Anakwah et al. (2024a) also found differential performance between recall and recognition/forced choice tasks by mock-witness interviews from a collectivistic culture and argued that individuals socialised in collectivistic cultures may be more acquiescent in such contexts. The response format may have altered the perceived pressure of being a research participant, increasing a perceived obligation to provide the ‘right’ response which provides more information, possibly emphasising ethical values that are culturally associated with the group of Chinese participants (e.g., Confucian role ethics, Buchtel et al., 2018) to a greater extent than for their UK counterparts. Another explanation is that the multiple-choice format for the follow-up interviewing questions led participants to engage in a recognition task which is less effortful than memory retrieval.

Secondly, while we did not find a significant effect of proximity of relationship with the mock-perpetrator on information disclosure in free reporting, descriptive statistics showed a pattern of lower reporting when there was a relationship with the mock-perpetrator vs. a stranger in spontaneous reporting. Moreover, the relational effect was present in the forced choice follow-up questions, with the same pattern of reporting indicating decreased information disclosure the closer the proximity of the relationship. Previous related research in investigative interviewing supports this finding (Nicksa, 2014; Schwartz & DeKeseredy, 1997; Tolsma et al., 2012) and has a strong foundation on evolutionary theories (Hamilton, 1964) and group affiliation (Brewer, 1979; Tajfel et al., 1971). Recent research has also explained that social relations affect our moral reasonings when judging others’ behaviour (Rai & Fiske, 2011). Importantly, our findings only partially align with Ding et al. (2025) who found these combined effects of affiliation and culture in moral judgements. In that sense, the lack of statistically significant evidence of this effect in the current research could be due to methodological differences.

Ding et al. (2025) used vignettes and ratings rather than behavioural measures, which limit ecological validity (Aguinis & Bradley, 2014), whereas the current research overcomes some of these limitations by measuring reporting behaviour in an online format. The differential strength in the influence of proximity of the relationship between spontaneous reporting and selecting forced choice answers in our research, as well as the partial discrepancy with Ding et al. (2025), could indicate that these effects are smaller as tasks become more complex due to competing demands. The research context puts participants in an ethical dilemma of cooperating to appease the sense of obligation to report incriminating information while at the same time navigating a strong moral compass of protecting a ‘known’ perpetrator in line with affiliation values (Berry et al., 2024; Buchtel et al., 2018). Regarding the latter, when anchored, internal values related to the affiliated group can also lead to harsher judgement of moral lapses by group members, coined as the ‘black sheep effect’, as in-group members are perceived as a threat to the group itself (Ellemers & Van der Toorn, 2015; Taylor & Hosch, 2004), which could explain why there is an influence, but only to a limited extent, to report less information when there is a relationship with the perpetrator.

Altogether this partial effect of proximity of relationship influencing witness reports could be interpreted using the witness cooperation decision model introduced by De La Fuente Vilar (2022) in which individual factors—such as a witness–perpetrator relationship—can compromise or motivate decisions to cooperate or not, initially and throughout an interview; thus, there is oscillation in an uncooperativeness–cooperativeness continuum. According to Alison et al. (2014), verbal communication strategies can be used to attempt to appear cooperative by uncooperative interviewees. Findings on the use of communication strategies used by participants in their free reports suggested evidence across conditions that participants provided a detailed report, but this was higher when the mock-perpetrator was a stranger without a shared culture. Conversely, strategically withholding information increased in the closest relationship, when the mock-perpetrator was close to the participant and from a shared culture. This finding suggests a balancing act of reporting not too much nor too little.

Additionally, participants provided in their own words their rationale for reporting (or not reporting) information. These findings show some alignment between participants’ intended reporting and actual disclosure, arguably supporting that witness *cooperation* can be understood as *willingness to report* and affects information disclosure (De La Fuente Vilar, 2022). While cooperation was not directly manipulated in this study, participants in the close–same culture condition reported attempting to increase provision of inaccurate information in order to portray innocence or displace blame from the mock-perpetrator who was close to them, which was expected (H2) and in line with previous research showing that affiliation increases the willingness to support a friend if they were a suspect in an investigation by giving an alibi for them, even if it is a false one (Kienzle & Levett, 2018; Marion & Burke, 2017). Past research has also shown that lack of cooperation overall decreases disclosure, and to a lesser extent, accuracy (De La Fuente Vilar et al., 2020).

### 4.1. Limitations and Future Directions

Cultural influences appear to have affected amount of disclosure to a greater extent relative to effects of the proximity of the relationship to the perpetrator. The observed patterns in reporting suggest that disclosure was in line (at least to some extent) with reporting more information about a perpetrator who is a stranger from a different culture across both samples compared to other conditions. Therefore, the current research may not have provided a sufficiently robust test of the effect of proximity to the perpetrator, or of its combined influence with culture. This may have been because the research scenario did not create sufficiently high stakes, or because the study lacked the statistical power to detect an effect that was smaller than expected, but still practically important in investigative contexts. Nevertheless, patterns in reporting suggest that the current research paradigm, where a tailored target perpetrator is selected based on participants’ self-reported relationships, did influence their intentions to disclose information. Given the operational importance of deepening our understanding of the relationship between culture and relationship proximity with the perpetrator, this paradigm could be used in future research with alternative (higher-stakes) scenarios and/or in conjunction with other methods to increase perceptions of immersiveness and plausibility. Importantly, it can be used in future research with a larger sample and a fully crossed experimental design, which could allow random allocation even in groups across conditions enabling higher statistical power and reliability of the analyses.

A few limitations of the current research are based on the sampling strategy and participant recruitment approach. The current experiment was conducted in a second language for part of the sample. Therefore, there is a possibility that some of the differences in reporting could be exacerbated by language effects. Research demonstrates that individuals provide less information when reporting events in a second language (Ewens et al., 2016). Even for individuals who are bilingual speakers there is evidence of a loss in detail when providing reports in the non-native language, although not in accuracy (Hu & Naka, 2023). Another potential limitation is that the current Chinese sample may not be culturally representative of Chinese people in general. Only Chinese participants with sufficient English proficiency were included in the study, and some participants were not based in China, which could lead them to follow a different communication style than the one established in the country of origin, perhaps due to acculturation effects (Anakwah et al., 2020b). Therefore, future work examining if the cultural differences we found replicate when participants report information in their native language and country of origin is warranted. However, it should also be noted that comparisons between translated accounts are not necessarily a panacea, given the potential for variation in translation and interpretation (see for example, Korkman et al., 2026). Moreover, a real-world consideration is that, in some instances in an English-speaking country, individuals who show proficiency in the English language who witness an incident may be interviewed at the scene in English, in order to obtain an initial account or if an incident may not require a follow-up investigation . Another individual difference to explore in future research is age, given the age differences across our samples. While past research on age effects in interviewing contexts highlight differences between age cohorts (see Wright & Holliday, 2007), to our knowledge, there is no evidence of differences within cohorts. Wright and Holliday (2007) included young adults aged 17 to 31 and found differences between young and old cohorts (aged 60 and above) explained by age-related episodic memory decline, which is not expected to affect those in the current sample (i.e., young adults). Lastly, it should be noted that the differences in reporting that were examined in this research were related to objective details given the opportunity of analysis against ground-truth, but future research could investigate how culture influences subjective and contextual comments in witness reports (de Bruïne et al., 2025).

Moreover, the current research only focused on comparisons between two cultural groups which are not monolithic (Hong et al., 2000) and are not the only expression of low- and high-context cultural communication preferences and individualistic–collectivist orientation. We encourage future research to continue developing the exploration of these differences both within and across diverse and different cultures. Similarly to past research (e.g., Anakwah et al., 2020b; Hope et al., 2023), we included measures of communication style, individualism–collectivism orientation and face-saving, where findings only partially supported the expected differences across conditions (see Supplementary Materials). So, we call again (see Hope et al., 2022, 2025) to critically review past dichotomous measures and concepts that fail to capture the complexity of differences across cultures (see also A. B. Cohen, 2009; Fiske, 2002). Additionally, when examining cultural variation, representation in the design along with a collaborative approach across equitable research partners are the ways forward to making insightful comparisons (Adams et al., 2015).

Another potential concern about this research is its limited ecological validity given the low stakes of the research scenario and given the online context. Future research is needed to examine how these patterns of reporting are displayed in a face-to-face interactive interview, rather than in a written report provided in an online context, which may have blurred some cultural norms (Hope et al., 2026; Wurtz, 2005). Given the rapid incorporation of online crime reporting platforms (Wells et al., 2023), it is important to empirically examine any benefits of online reporting which strips the traditional social context of investigative interviews, and particularly to consider any potential detrimental effects for individuals with high-context communication orientations. However, elements of potential effects due to cross-cultural differences between interviewer and interviewee (Beune et al., 2010; Hope et al., 2022) should be considered with more attention in face-to-face interactions. Power distance and relationship to authority should be taken into account in this line of future research as interactions with police, even in simulated investigative interviewing experiments, can exacerbate cultural differences in reporting (Anakwah et al., 2024b). On a related vein, future research is also needed to investigate if this paper’s findings replicate beyond the witness context developed into suspect and human intelligence gathering interviewing settings.

While the proposed methodology is more interactive than existing research using vignette methodologies and has proved to be effective to manipulate the proximity between a mock-witness and a perpetrator, this is only one aspect that can affect interviewee cooperation. The influence of the length and type of relationship between them could be examined in future research. Alternative experimental manipulations for cooperation should also be considered (see Brimbal et al., 2019; Houston & Russano, 2026) given the effect it can have on disclosure (De La Fuente Vilar et al., 2020). In addition, the influence of the types of crimes needs further exploration, as it likely moderates the extent to which individuals withhold incriminating information about perpetrators close to them (Nicksa, 2014). As discussed above, motivations to be loyal may be offset by the moral command to cooperate with the police when placed in this ethical dilemma (Berry et al., 2024). Lastly, a replication of this crime type with a different stimulus is also warranted. Using videos that depict the lived-in experience of the participants in each cultural context is advised to avoid a potential confound (e.g., Anakwah et al., 2020b).

### 4.2. Conclusions

This research demonstrates the importance of considering the role of culture in investigative interviewing as there are differential disclosure effects that can be detrimental to investigative outcomes. Our research replicates previous findings showing that individuals socialised in collectivistic cultures with a high-context communication style may spontaneously report less information than what is available to them (Anakwah et al., 2020a, 2020b; de Bruïne et al., 2025; Hope et al., 2023; Weiss et al., 2025). This pattern of less detailed reporting by witnesses from a high-context communication and a collectivistic culture can be exacerbated in cross-cultural settings in which meaning is not read ‘between the lines’ and communication errors can occur (Hope et al., 2026). In this research, this finding may be further exacerbated by the online format of reporting information that strips away some of the contextual and non-verbal cues that are used in high-context communication (Wurtz, 2005).

Furthermore, we add to this body of research novel empirical evidence of detrimental effects on disclosure due to a relationship between witness and perpetrator and cautiously note that cultural differences may also influence disclosure rates. These findings have important implications for cross-cultural interviewing and investigative contexts more broadly, where interviewers may need to gain cooperation from an interviewee who may be reluctant to disclose incriminating information about a perpetrator due to existing personal relationships or group affiliation. If there is an underlying culturally based influence on information reporting and more detail is necessary, interviewers can provide instructions to set reporting expectations in order to bridge the gap (Kontogianni et al., 2026) and make relevant adaptations to rapport-building (e.g., Hope et al., 2025; Ng et al., 2023), which are crucial to facilitate cooperation (Gabbert et al., 2021), especially with uncooperative witnesses (De La Fuente Vilar, 2022). 

## Figures and Tables

**Table 1:** Means and standard deviations for use of communication strategies in free report.

	Cultural Group		Proximity of the Relationship		
	UK	China	Close–Same Culture	Stranger–Same Culture	Stranger–Different Culture
	*M* (*SD*)	*M* (*SD*)	*M* (*SD*)	*M* (*SD*)	*M* (*SD*)
Providing a detailed report	5.71 (1.32)	5.73 (1.36)	5.17 (1.53) _c_	5.77 (1.15) _c,d_	5.95 (1.24) _d_
Only partial information	2.97 (1.83) _a_	3.69 (2.15) _b_	4.34 (1.87) _c_	2.32 (1.22) _d_	3.19 (2.11) _d_
Providing known information	2.95 (1.49)	3.36 (1.41)	3.32 (1.33)	3.19 (1.33)	3.06 (1.56)
Giving short answers	2.34 (1.01)	2.24 (1.02)	2.51 (1.03)	2.36 (0.92)	2.18 (1.03)

*Note*: Means sharing a common subscript are not statistically different at *p* < 0.05 according to corrected post hoc comparisons.

**Table 2:** Self-reported communication strategies used in free reporting by condition.

	Cultural Group		Proximity of the Relationship		
	UK	China	Close–Same Culture	Stranger–Same Culture	Stranger–Different Culture
Gave all the information/all true information	0.08	0.13	0.04	0.07	0.10
2. Gave the information they could remember/that was witnessed not heard/from memory/chronologically	0.29	0.24	0.10	0.11	0.33
3. Omitted information (believed to be known/unnecessary) and provided overview/key points only	0.07	0.04	0.06	0.01	0.04
4. Provided inaccurate information to make known person look innocent or blamed the other driver	0.02	0.02	0.07	0.01	0.03
5. Provided important/incriminating information that may help the police due to the incident severity	0.08	0.02	0.03	0.02	0.05
6. No reported strategy	0.00	0.01	0.00	0.00	0.01

*Note*: Values do not add up to 100% as participants reported using multiple strategies.

## Data Availability

The original data presented in the study are openly available in the Open Science Framework (https://osf.io/592re/overview?view_only=8c48b6fe5f8048b2b922a8aa7dcc4b43 accessed on 24 August 2026).

## Note

1 The term *Coloured* refers to people of mixed race in the South African context (Adhikari, 2009 as cited in Weiss et al., 2025) and is used by people to describe their cultural identity as well as officially in the national census.

## Supplementary Materials

The following supporting information can be downloaded at https://www.mdpi.com/article/10.3390/bs16091540/s1.
