# Supplementary material for: Culture of Silence: Examining the Influence of Culture in Mock-Eyewitness Reports
Source: Behav Sci (Basel). 2026 Sep 1;16(9):1540. doi: 10.3390/bs16091540 (PMC13603823; doi:10.3390/bs16091540)
Supplement: Supplementary file 1 [file behavsci-16-01540-s001.zip › behavsci-4355865-supplementary.pdf]

Culture of Silence?: Examining Cultural Differences and Cooperation in Eyewitness Reports

Supplementary Materials

Method

Video Stimulus

The video depicts a white taxi that has stopped in the middle of the road and a black car trying to drive away after it hit some cars. The driver of the black car that caused the accident is not visible to the participant. The taxi driver is outside his vehicle demanding insurance information from the driver in the black car. Next, the black car manoeuvres erratically in an attempt to flee the scene whilst causing the traffic to stand still on both lanes. The taxi driver then is standing in the middle of the street in front of the black car impeding its movement and then hits the driver's window with a stick. Afterwards, the black car moves forward hitting a second white taxi on the opposite lane. After that impact, the black car reverses and hits another red car parked in the rear. Next, the black car hits the first taxi again, and another SUV vehicle while escaping straight down the street accelerating.

Results

Exploratory Analyses

Communication Strategies by Condition

A series of two-way ANOVA were conducted to examine the main effects and the combined effect of Cultural Group x Proximity of the Relationship on the use of the first strategy, giving detailed information. There was a non-significant main effect of Cultural group, $F(1, 157) = 0.84$, $p = .361$; and a significant effect of proximity of the relationship, $F(2, 157) = 5.13$, $p = .007$, $\omega^2 = 0.05$. The interaction effect was also non-significant, $F(1, 157) = 0.80$, $p = .450$. Post-hoc tests with Bonferroni corrections revealed a significant difference, $p = .005$, $d = 0.62$, 95% CI (1.10, 0.14), in participants' use of the 'providing detailed information' strategy, with lower use of this strategy by participants in the Close–

Same Culture (*M* = 5.17, *SD* = 1.53) in comparison to participants in the Stranger–Different Culture condition (*M* = 5.95, *SD* = 1.24). The use of providing detailed information in the Stranger–Same Culture (*M* = 5.77, *SD* = 1.15) was not statistically different to the other conditions, all *p*s > .05.

Providing only partial information was the second strategy examined. Visual examination of the Q-Q plot showed a non-normal distribution. Levene's test for equality of variance was significant (p < .001). As Welch corrections cannot be applied in the two-way ANOVA, individual tests were conducted to examine the effects of Cultural Group and Proximity of the relationship on the use of the strategy: 'providing only partial information'. There was a significant main effect of Cultural group, Welch *t* (151.77) = - 2.32, *p* = .022, *d* = 0.36, 95% (-1.35, -0.11), indicating a significantly lower use of this strategy by UK (*M* = 2.97, *SD* = 1.83) participants vs their Chinese counterparts (*M* = 3.69, *SD* = 2.15). There was also a significant effect of proximity of the relationship on the use of this strategy, Welch corrected *F*(2, 84.26) = 15.31, *p* < .001, ω^2 = .10, 95% CI [0.03, 0.19]. Games-Howell post-hoc comparisons with Tukey corrections revealed a significantly higher use by participants in the Close–Same Culture (*M* = 4.34, *SD* = 1.87) condition, in comparison to the Stranger–Same Culture (*M* = 2.32, *SD* = 1.22) condition, *p* < .001, *d* = 1.06, 95% CI [.46, 1.65]; as well as against the Stranger–Different Culture condition (*M* = 3.19, *SD* = 2.11), *p* = .004, *d* = 0.60, 95% CI [0.14, 1.07].

The third strategy was 'providing known information' and it also failed the homogeneity tests (Levene's *p* < .001). The same corrections as in with the previous strategy were applied. There was a non-significant main effect of Cultural group, Welch *t* (160.86) = -1.79, *p* = .075, and a non-significant effect of proximity of the relationship on the use of this strategy, Welch corrected *F*(2, 75.28) = 0.50, *p* = .610.

The last strategy analysed was ‘giving short answers’. There was a non-significant main effect of Cultural group, F (1, 157) = 0.27, p = .602, and a non-significant main effect of Proximity of the relationship on the use of this strategy, F (2, 157) = 1.51, p = .225.

Qualitative Data Analysis of Self-Reported Communication Strategies

Most participants reported using at least one strategy (n = 142, 87%), but some used two (n = 19, ~11%), and one participant (n = 1, less than 1%) reported using three different strategies and another one reported not using a strategy (n = 1, less than 1%). Overall, most strategies were used by participants in the UK sample (55% vs 45% China). Many strategies received very low frequencies.

Face Saving Needs and Cultural Group

To further understand the effect of Cultural group on face saving needs, comparisons were run in the different subscales (Park et al., 2012). There was a significant difference in scores for Self-Negative Face (Cronbach’s α = .75), Welch t (159.15) = - 3.19, p = .002, d = -0.50 (95% CI -0.81, -0.19). Participants from China (M = 4.22, SD = 0.62) average scores were significantly higher than their UK counterparts (M = 3.87, SD = 0.76). No significant differences were found between the groups in scores measuring Other-Positive Face (Cronbach’s α = .77, p = .573), Self-Positive Face (Cronbach’s α = .79, p = .401), and Other-Negative Face (Cronbach’s α = .72, p = .063). An ANCOVA was ran to control for the effect of Self-Negative Face specifically, and Cultural group maintained its effect on free recall reporting, F(1, 160) = 42.55, $p < .001$, $\omega^2 = .20$, 95% CI [0.10, 0.31].

Individualism-Collectivism and Cultural Group

To examine differences by Cultural group on Individualism and Collectivism, we first analysed the reliability of the measure by Triandis & Gelfand (1998), which was acceptable to good (Field, 2018) across the four subscales: Horizontal Individualism (Cronbach’s α =

.79), Vertical Individualism (Cronbach's α = .76), Horizontal Collectivism (Cronbach's α = .71), and Vertical Collectivism (Cronbach's α = .85).

Given the significant differences across Cultural Group on two of the Individualism-Collectivism subscales, and the significant effect of Cultural Group on the key dependent variable, we set to examine whether cultural group affects reporting by running individual ANCOVAs. There was a significant effect of Cultural Group after controlling for the general effect of Vertical Individualism, $F(1, 160) = 42.38, p < .001, \omega^2 = .20$, 95% CI [0.10, 0.31]. Similarly, there was a significant effect of Cultural Group after controlling for the general effect of Horizontal Collectivism, $F(1, 160) = 38.39, p < .001, \omega^2 = .19$, 95% CI [0.09, 0.30].

Cultural Differences on Perceptions of the Police

Lastly, cultural differences in perceptions of the police were analysed. A composite score measuring perceptions of the police using Tyler and Fagan's (2008) scale was used as the dependent variable, which showed excellent reliability (Cronbach's Alpha .91; Field, 2018). There were no significant differences, $t\,(161) = -\,1.36, p = .175, d = -\,0.21$, 95% CI [-0.52, 0.10], in perceptions of the police by individuals from the UK ($M = 3.12$, $SD = 0.54$) and from China ($M = 3.23$, $SD = 0.55$). No follow up tests were conducted given this initial non-significant difference.

Discussion

Measurement Challenges for Cultural Orientation

Direct communication style and face-saving needs were not found to be different across cultural groups and their effect on free reporting was not found to be significant. Additionally, while there were differences across the conditions on two individualism-collectivism orientations, these were not in line with expectations for individuals from these

cultures. An explanation for these findings is that these scales are limited in their ability to capture the concepts they aim to measure (see Cohen, 2009; Fiske, 2002).
